# Mediolateral/lateral episiotomy with operative vaginal delivery and the risk reduction of obstetric anal sphincter injury (OASI): A systematic review and meta-analysis

**DOI:** 10.1007/s00192-022-05145-1

**Published:** 2022-04-15

**Authors:** Nicola Adanna Okeahialam, Ka Woon Wong, Swati Jha, Abdul H. Sultan, Ranee Thakar

**Affiliations:** 1 Urogynaecology Clinical Research Fellow, Croydon University Hospital, Croydon, UK; 2 Urogynaecology Subspecialty Registrar, Croydon University Hospital, Croydon, UK; 3 Consultant Obstetrician and Urogynaecologist, Department of Urogynaecology, Sheffield Teaching Hospitals, Sheffield, UK; 4 Consultant Obstetrician and Urogynaecologist, Croydon University Hospital, Croydon, UK; 5 St George’s University of London, London, UK

**Keywords:** Mediolateral episiotomy, Lateral episiotomy, Obstetric anal sphincter injury, Operative vaginal delivery

## Abstract

**Introduction and hypothesis:**

OASI complicates approximately 6% of vaginal deliveries. This risk is increased with operative vaginal deliveries (OVDs), particularly forceps. However, there is conflicting evidence supporting the use of mediolateral/lateral episiotomy (MLE/LE) with OVD. The aim of this study was to assess whether MLE/LE affects the incidence of OASI in OVD.

**Methods:**

Electronic searches were performed in OVID Medline, Embase and the Cochrane Library. Randomised and non-randomised observational studies investigating the risk of OASI in OVD with/without MLE/LE were eligible for inclusion. Pooled odds ratios (OR) were calculated using Revman 5.3. Risk of bias of was assessed using the Cochrane RoB2 and ROBINS-I tool. The quality of evidence was assessed using Grading of Recommendations Assessment, Development and Evaluation (GRADE).

**Results:**

A total of 703,977 patients from 31 studies were pooled for meta-analysis. MLE/LE significantly reduced the rate of OASI in OVD (OR 0.60 [95% CI 0.42–0.84]). On sub-group analysis, MLE/LE significantly reduced the rate in nulliparous ventouse (OR 0.51 [95% CI 0.42–0.84]) and forceps deliveries (OR 0.32 [95% CI 0.29–0.61]). In multiparous women, although the incidence of OASI was lower when a ventouse or forceps delivery was performed with an MLE/LE, this was not statistically significant. Heterogeneity remained significant across all studies (I^2^ > 50). The quality of all evidence was downgraded to “very low” because of the critical risk of bias across many studies.

**Conclusions:**

MLE/LE may reduce the incidence of OASI in OVDs, particularly in nulliparous ventouse or forceps deliveries. This information will be useful in aiding clinical decision-making and counselling in the antenatal period and during labour.

**Supplementary Information:**

The online version contains supplementary material available at 10.1007/s00192-022-05145-1.

## Introduction

Operative vaginal delivery with either ventouse or forceps is used to facilitate delivery for a number of maternal and foetal indications [1]. In the UK, operative vaginal delivery is the method of delivery in 12% of women [2]. A worldwide survey of operative vaginal delivery practice in the 1990s demonstrated that forceps were widely used in English-speaking countries such as the USA, UK, Ireland, New Zealand, Canada and Australia. However, ventouse deliveries were widely used in countries within Northern Europe, Africa, the Middle East and Far East countries including China, Hong Kong, Japan, Korea and Thailand [3]. There has been a reduction in forceps use in a number of countries such as the USA, which reduced their rate from 5.1% to 0.6% (1990–2015) [4]. Moreover, in Sweden and Austria, the rate of forceps use has reduced from 1% to 0% (2005–2016) [5]. However, in units in the UK, the incidence of forceps is increasing [6]. Obstetric anal sphincter injury (OASI) occurs in approximately 6% of first vaginal births [7]. This risk is increased further with operative vaginal deliveries, in particular forceps-assisted deliveries.

OASI is a significant risk factor in the development of anal incontinence, with significant implications for the quality of life. Therefore, identification of modifiable risk factors to prevent OASI is important [8, 9]. An episiotomy can be used to increase the dimensions of the vaginal outlet and to create a controlled incision in the perineal body away from the anal sphincter [10]. Regarding OASI incidence, lateral episiotomy (LE), which begins 1–2 cm away from the midline, has been shown to not differ significantly from a mediolateral episiotomy (MLE) [10, 11]. The Royal College of Obstetricians and Gynaecologists (RCOG) Green Top Guideline for assisted vaginal birth [1] acknowledges that the evidence to date supporting the use of MLE at operative vaginal delivery, in terms of preventing OASI, is stronger for nulliparous women and for birth via forceps. However, it is stated that in the absence of robust evidence to support either routine or restrictive use of episiotomy at assisted vaginal birth, the decision should be tailored to the circumstances at the time and the preferences of the woman [1]. Yet, the RCOG Green Top Guideline for the management of OASI [12] advises that MLE should be considered with assisted vaginal birth. This lack of clarity has caused confusion amongst professional [13] and patient groups [14]. To date no meta-analysis has been performed to investigate the effect of MLE/LE with forceps deliveries and OASI incidence. Two meta-analyses have evaluated MLE/LE use with ventouse deliveries [15, 16]. However, the results of these reviews were conflicting [15, 16]. Sagi-Dain et al. [16] found a non-significant decrease in the incidence of OASI with MLE and suggested that MLE may be harmful in parous women, whilst Lund et al. [15] demonstrated a significant reduction in the incidence of OASI with MLE. In addition, neither review evaluated the effect of MLE/LE with forceps deliveries on OASI incidence. Therefore, up-to-date evidence is required to address these inconsistent findings.

The aim of this study was to investigate the effect of MLE/LE use with operative vaginal delivery on the risk of OASI.

## Materials and methods

This systematic review of the literature was conducted in accordance with the Preferred Reporting Items for Systematic Reviews and Meta-Analyses (PRISMA) guidelines [17]. Meta-analysis of Observational Studies in Epidemiology (MOOSE) guidelines for reporting meta-analyses of observational studies were also followed (Appendix S1) [18]. A protocol was developed and can be reviewed in the international prospective register of systematic reviews (PROSPERO) register (CRD 42020196579) [19]. Our primary research question was: “Does MLE/LE use with operative vaginal delivery reduce the risk of obstetric anal sphincter injury in comparison to no episiotomy?”. A PICO approach was followed:

- **Population:** Nulliparous and multiparous women undergoing operative vaginal delivery
- **Intervention:** MLE/LE
- **Comparator:** No episiotomy
- **Outcome:** OASI

OVID Medline, Embase and the Cochrane Library from inception to June 2020 were searched using the terms “anal sphincter injury”, “episiotomy”, “instrumental”, “forceps” and “vacuum”, including medical subject headings (meSH) terms, with no restriction on language or year of publication. A manual search of references from identified studies was also conducted to identify other relevant studies. Studies were included if the episiotomy was a MLE or LE. Studies reporting the use of MLE/LE with spontaneous vaginal births or midline episiotomy were excluded. Other relevant systematic reviews of MLE/LE with operative vaginal delivery and the reference lists of the eligible studies were also searched [15, 16]. A full search strategy can be found in the electronic supplementary material (Appendix S1). Results were exported to Zotero reference management system and de-duplicated. Randomised controlled trials (RCTs), non-randomised controlled trials, prospective and retrospective observational studies analysing the risk of OASI in women undergoing operative vaginal delivery with and without MLE/LE were eligible for inclusion. Case reports, case series, narrative reviews and conference abstracts were excluded. A full list of excluded studies is given in Table S1.

Two authors (N.A.O., K.W.W.) independently screened the titles and abstracts of all retrieved studies to obtain studies for full-text assessment. Any disagreements surrounding eligibility for full-text assessment were resolved by the senior reviewers or through consensus-based discussion. Full-text articles which met the inclusion criteria were then assessed by the two authors. Following this, the authors independently collected data from eligible studies, using a standardised electronic data extraction form. This included data regarding operative vaginal delivery, study characteristics, parity, type of operative vaginal delivery, type of episiotomy and rate of OASI. Translations were sought for any study not in English. Authors of included studies were contacted if the full text could not be retrieved and if the data reported were incomplete, unclear or published in a manner that was not extractable. If the author did not respond, unpublished data provided by the same author from the previously published systematic review of the risk of OASI with MLE/LE and ventouse delivery were used [15].

Review Manager 5.3 (The Cochrane Collaboration) and Meta-Essentials (version 1.5) [20] were used to analyse data. Data were reported as odds ratios (OR) and their corresponding 95% confidence interval (95% CI) bounds. The heterogeneity amongst studies was calculated using the I^2^ statistic. An I^2^ > 50 % was considered as significant heterogeneity and I^2^ > 80 was considered as very significant heterogeneity. Meta-analysis was performed if each outcome was represented in at least two studies, using the fixed-effects (Mantel-Haenszel) or the random-effects (DerSimonian and Laird) model. The random-effects model was used if heterogeneity was significant (I^2^ > 50 %). Sensitivity analysis for the primary outcome was conducted by removing high/critical bias studies to assess for methodological heterogeneity. Subgroup analyses were then performed to determine potential sources of clinical heterogeneity by separating participant data into sub-groups deemed to be categorical predictors, such as parity and instrument type. A *p*-value < 0.05 was considered statistically significant. Presence of publication bias was assessed using a funnel plot and Egger’s regression analysis.

Risk of bias assessment of RCTs was conducted using the Cochrane risk-of-bias tool for randomized trials (RoB 2) [21]. Non-randomised studies, including observational studies, were assessed using the Risk Of Bias in Non-randomized Studies-of Interventions (ROBINS-I) tool [22]. Risk of bias was assessed at an outcome level (not individual study level). Two reviewers independently assessed the overall quality of the evidence using criteria recommended by the Grading of Recommendations Assessment, Development and Evaluation working group (GRADE) [23]. Any disagreements surrounding eligibility for overall study quality were resolved by the senior reviewers or through consensus-based discussion. From the GRADE table, the difference between the anticipated absolute effect and 95% CI was used to calculate the number needed to treat (NNT) with its 95% CI [24]. No funding was required to complete this review.

## Results

**Fig. 1:**
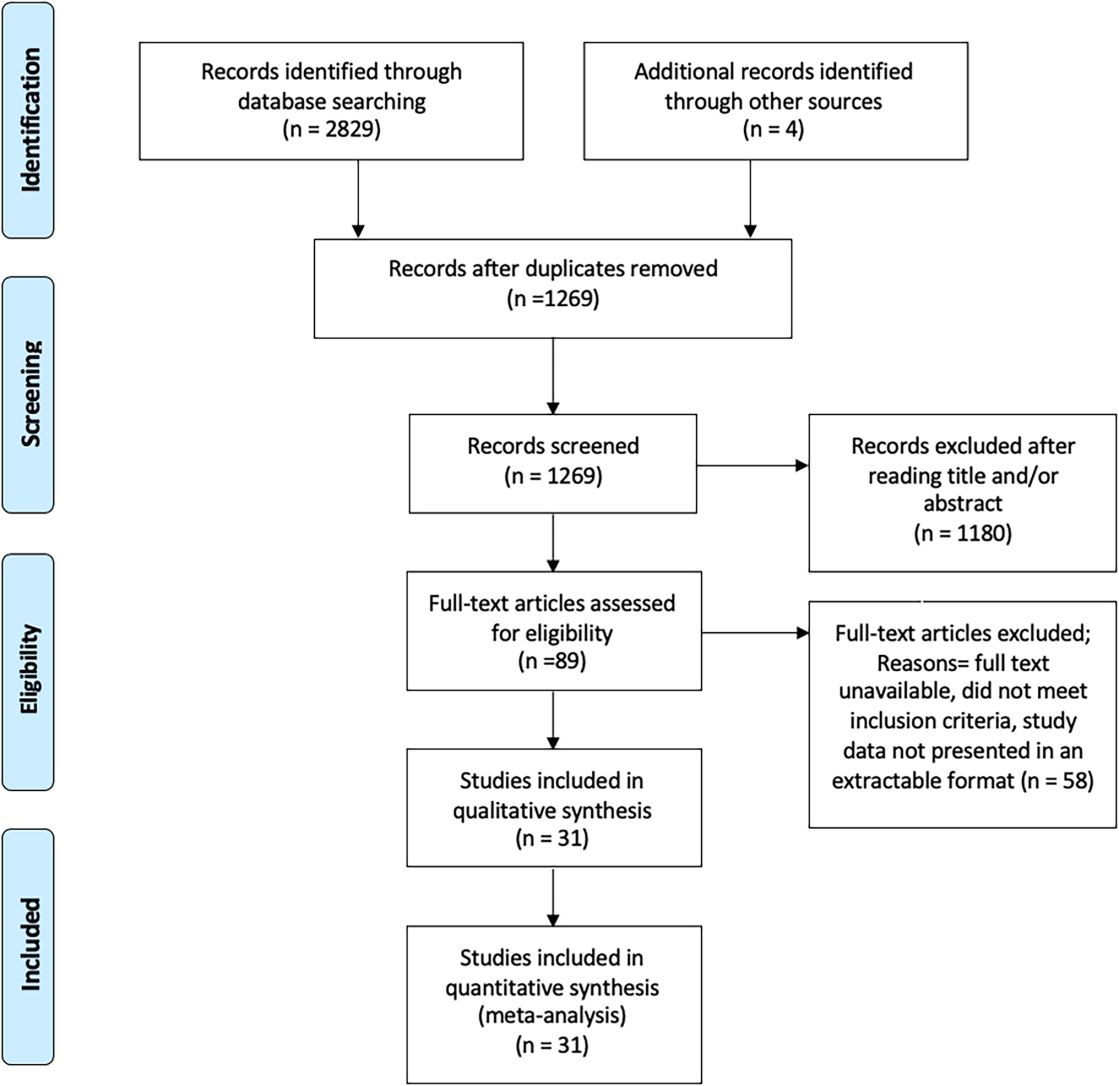
PRISMA flow diagram of the study selection process

Of the 1269 articles initially identified by the search, 89 were selected for full-text review. Thirty-one studies were eligible for inclusion and included in the meta-analysis (Fig. 1). Table 1 presents a detailed overview of the studies included in the meta-analysis. Two RCTs were identified [25, 26]. Other studies included six prospective observational studies [27–32] and 23 retrospective observational studies [33–55]. Overall risk of bias for the two RCTs [25, 26] was high. In 24 of the observational studies [28, 32–36, 38–55], overall risk of bias was critical. In one observational study, overall risk of bias was serious [37], and in the remaining four observational studies, overall risk of bias was moderate (Table S2, S3) [27, 29–31] .

**Table 1:** Overview of included studies

Authors (year)	Study type	Episiotomy type	Episiotomy incidence (%)	Parity	Instrument type	OASI incidence (%)	OR [95% CI]
Ampt et al. [54] (2013)	Retrospective case control	MLE	55.2	All- separated	Both-separated	7.2	1.07 [1.02, 1.14]
Aukee et al. [34] (2006)	Retrospective case control	MLE	84.0	All- pooled	Ventouse	3.8	0.36 [0.13, 0.98]
Baghurst et al. [35] (2012)	Retrospective cohort	MLE	55.0	All- separated	Both-separated	7.5	1.12 [0.96, 1.31]
Bodner-Adler et al. [36] (2018)	Retrospective cohort	MLE	65.0	Nulliparous	Ventouse	6.8	0.60 [0.31, 1.16]
Boujenah et al. [37] (2019)	Retrospective cohort	MLE	76.9	Nulliparous	Both-separated	2.8	0.38 [0.20, 0.74]
D'Souza et al. [38] (2020)	Retrospective cohort	MLE	81.9	Multiparous	Both-separated	6.0	0.08 [0.01, 0.51]
De Leeuw et al. [39] (2008)	Retrospective cohort	MLE	82.2	All- separated	Both-separated	3.4	0.14 [0.12, 0.16]
De Parades et al. [28] (2004)	Prospective cohort	MLE	95.7	Nulliparous	Forceps	12.9	0.42 [0.04, 4.43]
De Vogel et al. [40] (2012)	Retrospective cohort	MLE	81.0	All- separated	Both-separated	5.7	0.18 [0.13, 0.25]
Gachon et al. [41] (2019)	Retrospective cohort	MLE	40.3	All- separated	Both-separated	7.4	0.38 [0.26, 0.55]
Gurol-Urganci et al. [33] (2014)	Retrospective cohort	MLE	76.1	Nulliparous	Both-separated	7.1	0.49 [0.48, 0.51]
Hamouda et al. [27] (2017)	Prospective cohort	MLE	58.2	All- pooled	Both-separated	3.9	1.08 [0.38, 3.10]
Jango et al. [42] (2014)	Retrospective cohort	MLE	28.7	Nulliparous	Both-separated*	13.7	0.67 [0.63, 0.72]
Levin et al. [43] (2020)	Retrospective cohort	MLE	78.0	Nulliparous	Ventouse	2.3	0.56 [0.35, 0.91]
Macleod et al. [29] (2008)	Prospective cohort	MLE	78.4	Nulliparous	Both-separated	9.9	1.44 [0.88, 2.34]
Marschalek et al. [44] (2018)	Retrospective cohort	MLE	72.5	Nulliparous	Both-separated	5.2	0.68 [0.60, 0.76]
Meyer et al. [53] (2020)	Retrospective cohort	MLE	74.1	All- separated	Forceps	2.5	1.80 [0.52, 6.26]
Murphy et al. [25] (2009)	RCT	MLE	72.0	Nulliparous	Both-separated†	10.9	4.79 [0.22, 105.30]
Parnell et al. [30] (2001)	Prospective case control	MLE	53.0	Nulliparous	Ventouse	21.0	0.74 [0.41, 1.34]
Räisänen et al. [45] (2012)	Retrospective cohort	LE	84.9	All- separated	Ventouse	3.0	0.47 [0.35, 0.64]
Räisänen et al. [46] (2009)	Retrospective cohort	LE	90.0	All- separated	Both-separated†	1.5	1.09 [0.87, 1.36]
Rognant et al. [47] (2012)	Retrospective cohort	MLE	85.0	All- pooled	Ventouse	2.2	1.97 [0.60, 6.48]
Rygh et al. [31] (2014)	Prospective cohort	MLE/LE	55.0	Nulliparous	Both-pooled	11.0	0.72 [0.60, 0.87]
Sagi-Dain [26] (2020)	RCT	MLE	49.6	Nulliparous	Ventouse	3.7	0.67 [0.11-4.12]
Schmitz et al. [44] (2014)	Retrospective case control	MLE	66.5	All- separated	Both-separated†	2.1	0.04 [0.00, 0.79]
Shmueli et al. [49] (2017)	Retrospective cohort	MLE	66.0	All- separated	Ventouse	1.5	1.73 [0.99, 3.04]
Van Bavel et al. [50] (2018)	Retrospective cohort	MLE	89.6	All- separated	Both-separated	4.2	0.19 [0.18, 0.19]
Van Roon et al. [32] (2015)	Prospective cohort	MLE	90.0	Nulliparous	Both-pooled	5.4	3.18 [1.39, 7.27]
Vathanan et al. [55] (2014)	Retrospective cohort	MLE	78.7	All-pooled	Both-separated	9.2	0.18 [0.13-0.25]
Yamasato et al. [51] (2016)	Retrospective cohort	MLE	3.7	All- pooled	Both-separated	21.7	0.65 [0.14, 3.09]
Youssef et al. [52] (2005)	Retrospective cohort	MLE	71.2	All- pooled	Both-separated	8.7	0.99 [0.54, 1.81]

RCT: randomised controlled trial

MLE- mediolateral episiotomy

L/E: lateral episiotomy

*Only data for ventouse deliveries reported, unable to retrieve crude data for forceps-assisted deliveries from authors

^†^Data not extractable, data retrieved from previous systematic reviews [14, 15] as unable to retrieve crude data from authors

**Fig. 2:**
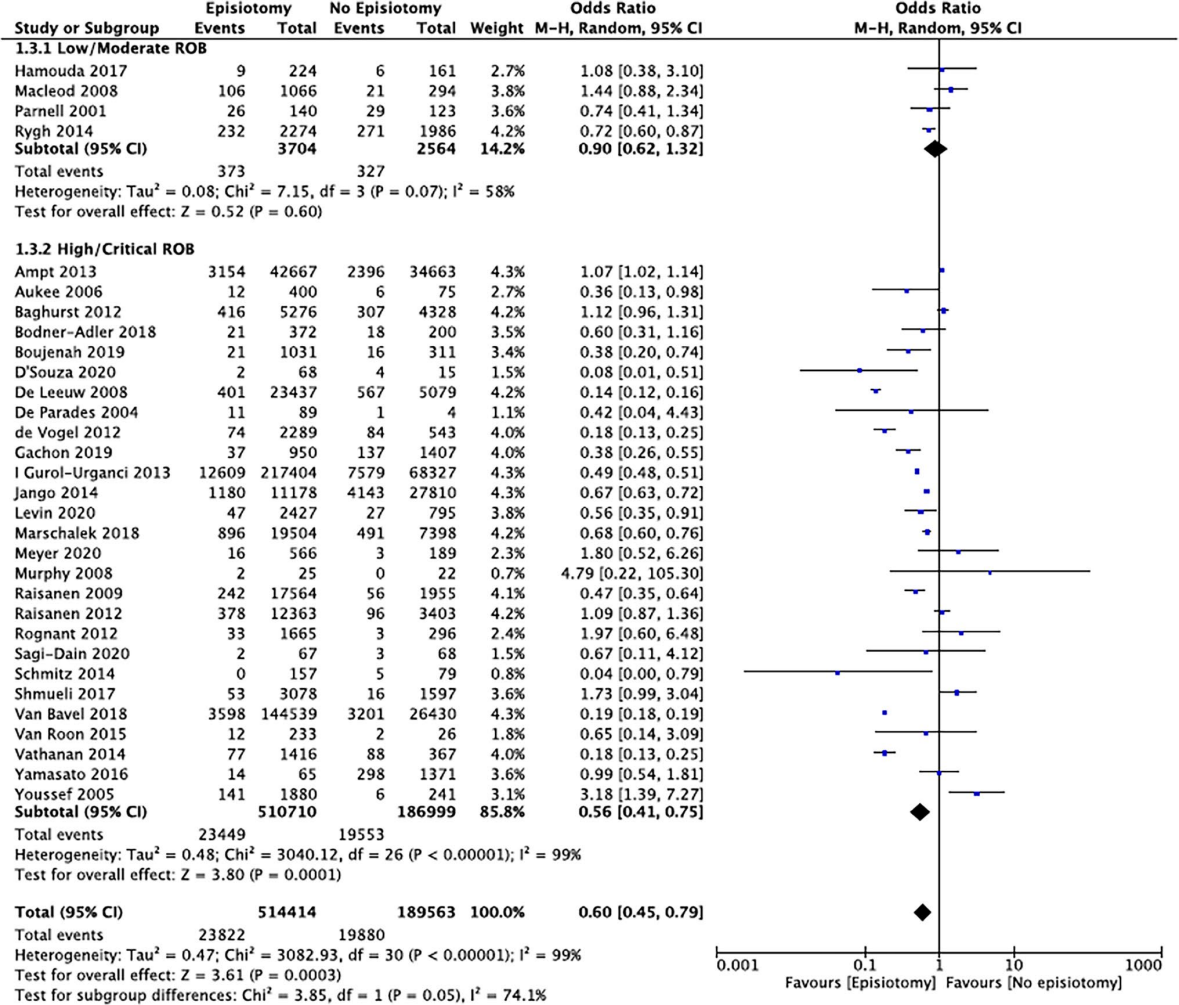
Risk of OASI in operative vaginal deliveries with or without episiotomy

Based on the inclusion criteria, 703,977 patients from 31 studies were included in this review for meta-analysis. MLE/LE was performed in an average of 69.0% (range 3.7–95.7%) of operative vaginal deliveries and OASI was diagnosed on average in 6.9% (range 1.5–21.7%) of cases. The meta-analysis showed a significant reduction in the OASI rate when operative vaginal deliveries were completed with an MLE/LE compared to deliveries without (OR 0.60 [95% CI 0.45–0.79]) (Fig. 2). The NNT was 26 (95% CI 18.2–50.0). On sensitivity analysis, there was no significant reduction in OASI rates (OR 0.90 [0.62–1.32]) in studies of low/moderate risk of bias. There was no strong evidence that the study risk of bias had an effect on the rate of OASI with or without MLE/LE (*p* = 0.05). Also, heterogeneity remained significant (low/moderate risk: I2 = 58%; high/critical risk: I2 = 99%).

**Fig. 3:**
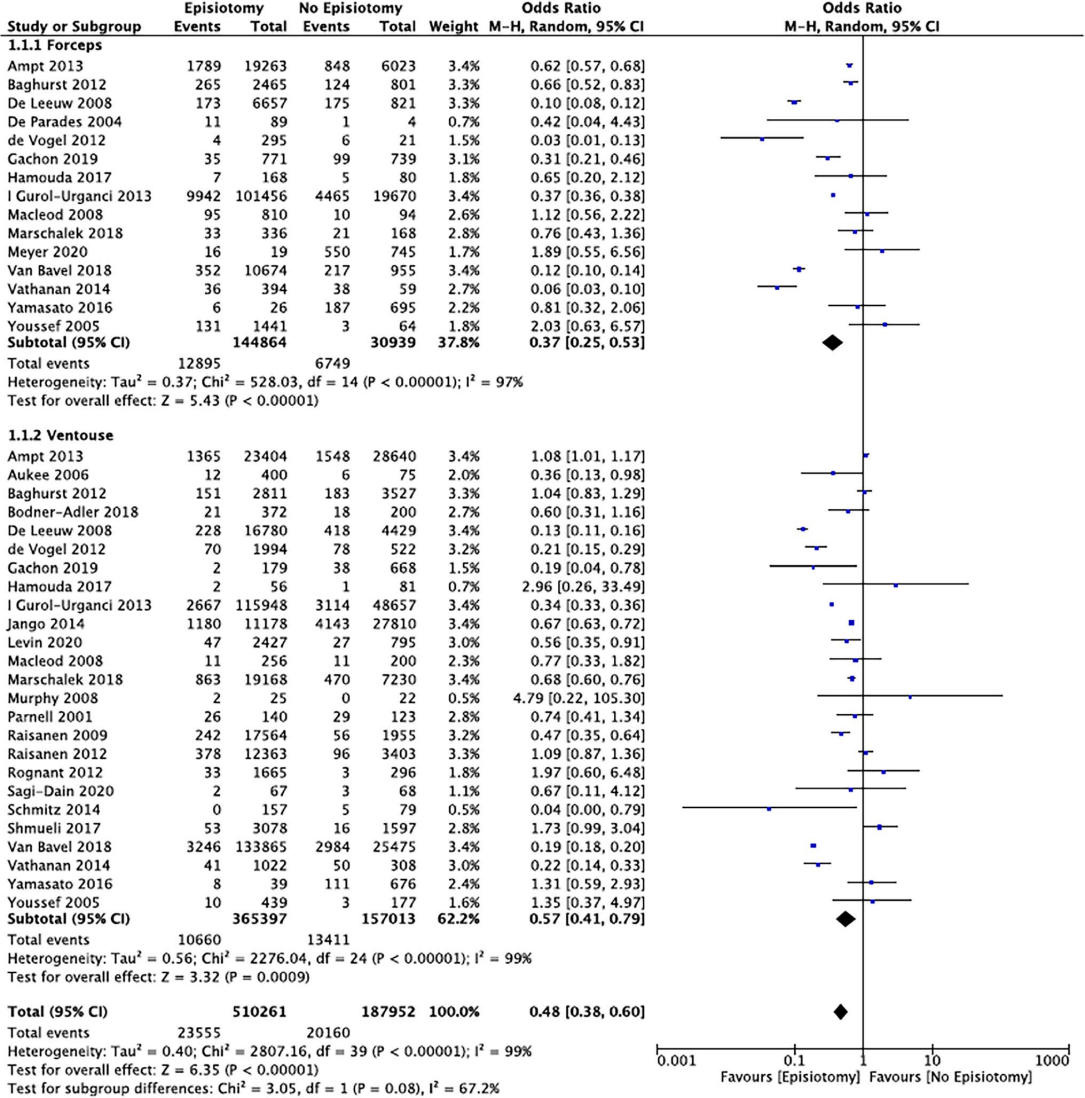
Risk of OASI in ventouse and forceps deliveries with or without episiotomy in nulliparous women

Sub-group analysis was completed for instrument type and parity. Data for ventouse deliveries were identified from 25 studies, forceps from 15 studies and 2 studies pooled all operative vaginal deliveries together. Of the 703,977 women, 74.2% (*n* = 522,410) had a ventouse delivery and 25.0% (*n* = 175,803) had a forceps delivery. MLE/LE was performed in an average of 64.4 % (range 4.3–90.0%) of ventouse deliveries and 77.3% (range 2.9–95.8%) of forceps deliveries. Meta-analysis showed a significant reduction in the rate of OASI when a ventouse (OR 0.57 [95% CI 0.41–0.79]) or forceps (OR 0.37 [95% CI 0.25–0.53]) was completed with an MLE/LE, compared to deliveries without (Fig. 3). The NNT for a ventouse delivery was 28 (95% CI 20.4–58.8), and for a forceps delivery it was 8 (95% CI 6.5–11.2). No statistically significant subgroup effect was found (*p* = 0.08) and heterogeneity remained very significant within each sub-group.

**Fig. 4:**
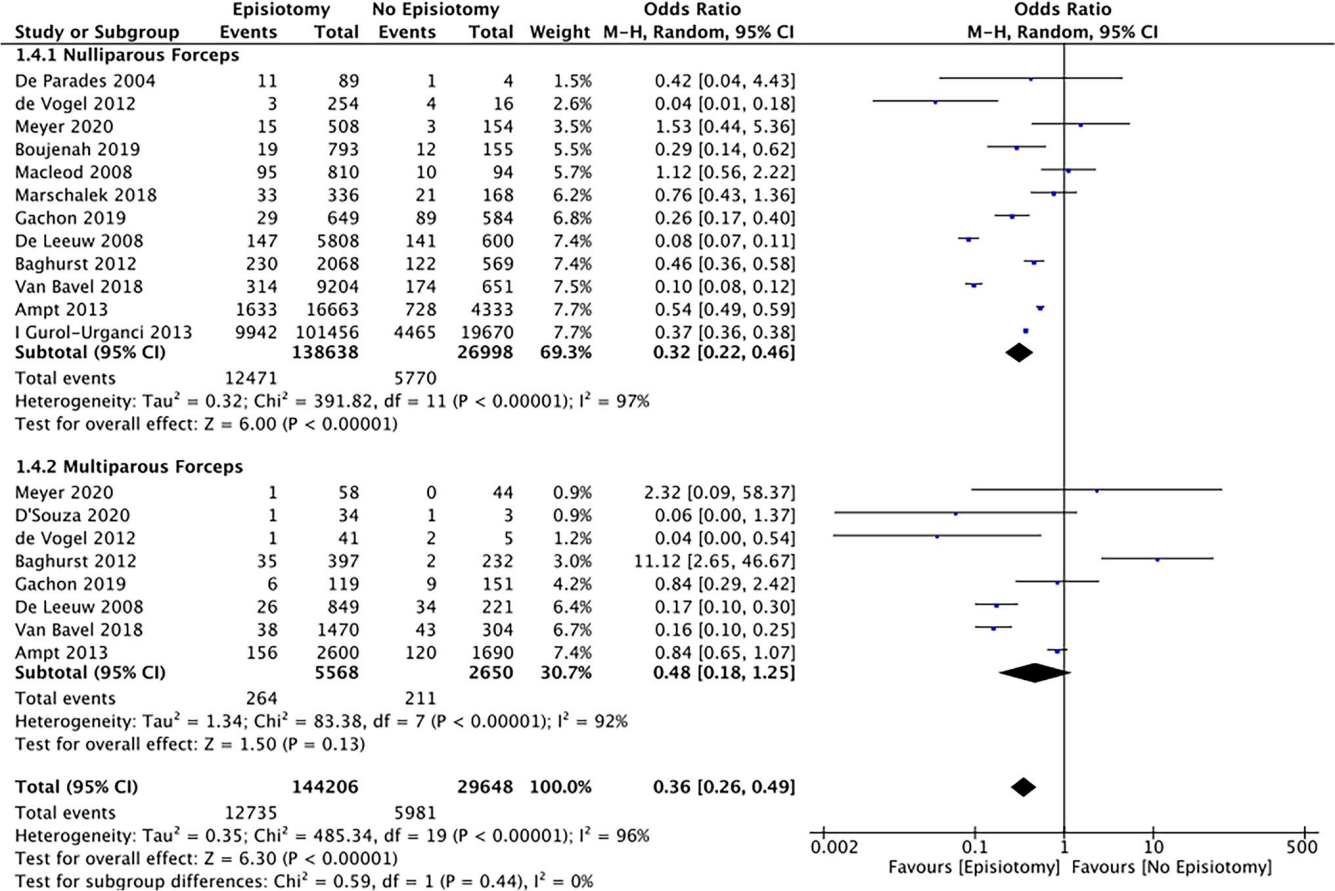
Risk of OASI in nulliparous and multiparous forceps deliveries with or without episiotomy

**Fig. 5:**
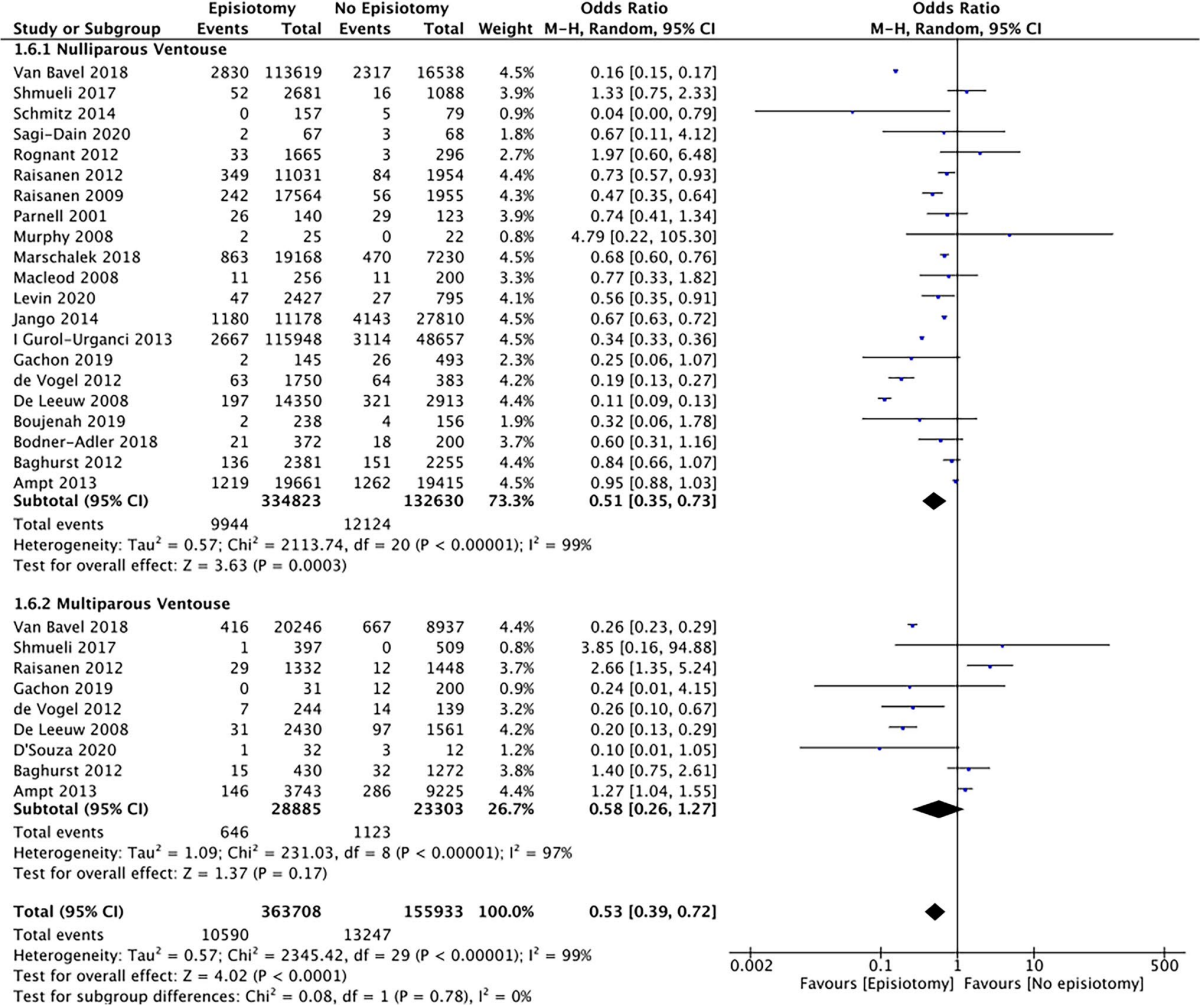
Risk of OASI in nulliparous and multiparous ventouse deliveries with or without episiotomy

Regarding parity, 633,089 (86.3%) women were nulliparous and 60,406 (7.8%) women were multiparous. Six studies pooled data from all women (*n* = 10,482) irrespective of parity undergoing operative vaginal delivery. In nulliparous women, the rate of OASI was significantly reduced when an MLE/LE was performed during a ventouse (OR 0.51 [95% CI 0.35–0.73]) or forceps (OR 0.32 [95% CI 0.22–0.46]) delivery (Figs. 4 and 5). In these women the NNT was 23 (95% CI 17.5–43.5) and 8 (95% CI 6.4–9.7) for a ventouse and forceps delivery respectively. However, in multiparous women, although the incidence of OASI was lower when an MLE/LE was performed with a ventouse or forceps delivery, this reduction did not reach statistical significance . The test for sub-group differences due to parity indicated there was no statistically significant subgroup effect (forceps [*p* = 0.44], ventouse [*p* = 0.78]). Despite sub-group analysis, heterogeneity remained very significant within each sub-group.

There was no publication bias amongst the included studies, as demonstrated by the symmetrical distribution of the funnel plot (Fig. S1). Egger’s regression analysis found no significant publication bias amongst the studies (*p* = 0.92). However, the quality of all evidence was downgraded to “very low” because of the critical risk of bias across many studies (Fig S3) and the very high level of heterogeneity (I2 value > 80%), which lowered the confidence in the estimate of effect. After review of the 95% CIs, evidence was also downgraded because of potential imprecision with the outcome estimates, considering the default minimal clinically important difference for dichotomous outcomes (0.8 to 1.25) [56]. The GRADE table is presented in Table 2.

**Table 2:** Overall quality of the evidence identified for meta-analysis

Outcome no. of participants (studies)	Relative effect (95% CI)	Anticipated absolute effects (95% CI)			Certainty
		With OASI	Without OASI	Difference	
Instrumental No. of participants: 703977 (31 observational studies)	OR 0.60 (0.47– 0.84)	10.5%	6.6% (5.0–8.5)	3.9% fewer (2.0–5.5)	⨁◯◯◯ Very low^a,b,c^
Forceps No. of participants: 175803 (15 observational studies)	OR 0.37 (0.25– 0.53)	21.8%	9.4% (6.5–12.9)	12.5% fewer (8.9–15.3)	⨁◯◯◯ Very low^a,b^
Ventouse No. of participants: 522410 (25 observational studies)	OR 0.57 (0.41–0.79)	8.5%	5.1% (3.7–6.9)	3.5% fewer (1.7–4.9)	⨁◯◯◯ Very low^a,b,c^
Nulliparous forceps № of participants: 165636 (12 observational studies)	OR 0.32 (0.22– 0.46)	21.4%	8.0% (5.6–11.1)	13.4% fewer –(10.315.7)	⨁◯◯◯ Very low^a,b^
Nulliparous ventouse № of participants: 467453 (21 observational studies)	OR 0.51 (0.35–0.73)	9.1%	4.9% (3.4–6.8)	4.3% fewer (2.3–5.7)	⨁◯◯◯ Very low^a,b^
Multiparous forceps № of participants: 8218 (8 observational studies)	OR 0.48 (0.18– 1.25)	8.0%	4.0% (1.5– 9.8)	4.0% fewer (1.8–6.4)	⨁◯◯◯ Very low^a,b,c^
Multiparous ventouse № of participants: 52188 (9 observational studies)	OR 0.58 (0.26– 1.27)	4.8%	2.9% (1.3– 6.0)	2.0% fewer (1.2–3.5)	⨁◯◯◯ Very low^a,b,d^

^a^Crucial limitation for one of more criteria substantial enough to lower one’s confidence in the estimate of effect.

^b^Very high level of heterogeneity (I2 value > 80%)

^c^95% confidence interval crosses 1 default minimally important difference (0.8 or 1.25)

^d^95% confidence interval crosses 2 default minimally important differences (0.8 and 1.25)

OR = odds ratio

CI = confidence interval

## Discussion

This meta-analysis of > 700,000 women showed that MLE and LE with operative delivery reduce the rate of OASI, particularly in nulliparous women. MLE/LE use in operative vaginal delivery was associated with a 40% reduction in the odds of OASI. In nulliparous women, an odds reduction of 49% and 68% was seen in ventouse and forceps deliveries with an MLE/LE respectively.

The main strength of our study is that it is the first meta-analysis reviewing outcomes following both ventouse and forceps delivery with MLE/LE in nulliparous and multiparous women. MLE and LE were combined as studies have demonstrated no difference in outcomes between the two types [10, 11]. In addition, it includes the largest number of nulliparous and multiparous women undergoing operative vaginal delivery. We conducted a comprehensive search with no language or date restrictions and contacted authors where possible to obtain unpublished data. We do acknowledge that there are limitations, particularly with the potential effect of the significant heterogeneity, although this was controlled for and explored further by using a random-effects model when pooling data for meta-analysis, sensitivity and sub-group analyses. However, there was inconsistent publication of adjusted odds ratios amongst the included studies, meaning unadjusted odds ratios were used for meta-analysis. Therefore, the unmeasured sources of confounding factors such as ethnicity, maternal age, birthweight and head circumference [57] may be a potential source of the significant heterogeneity between studies.

The risk of OASI is also associated with the angle at which an episiotomy is performed. A MLE should be performed at an angle of 60° from the midline, at crowning of the foetal head, subsequently resulting in a post-delivery angle of 45° [1, 11]. The incidence of OASI with MLE has been shown to reduce by 50% for every 6° of the MLE sutured angle away from the midline [58]. The angle of episiotomy was only measured in one study [32], where the EPISCISSORS-60® [59] were used. These are designed to cut at an angle of 60° and have been shown to produce an optimal post-delivery angle of 43°, meaning in this study, episiotomies were truly mediolateral. A prospective study by Andrews et al. [60], which investigated the practice of MLE amongst doctors and midwives, found that no midwife and only 22% of doctors performed a MLE at the desired angle. In addition, one-third of episiotomies performed by midwives were actually midline. Midline episiotomy, particularly in the context of operative vaginal delivery, significantly increases the risk of OASI in both nulliparous and parous women [61]. Consequently, if many of the episiotomies in the studies included in our meta-analysis were not truly mediolateral, the incidence of OASI might potentially be falsely high.

Another limitation of this study is that the meta-analysis included non-randomised studies. However, to date only two RCTs have been published. One only evaluated the effect of MLE in ventouse alone [26] and the other did not reach adequate statistical power [25]. The design of the latter study was a multicentre pilot study which demonstrated that an RCT of routine versus restrictive use of episiotomy at operative vaginal delivery is feasible. The sample size was limited by the ethical difficulties and time constraints involved in recruiting women to studies of emergency procedures in the second stage of labour. It can be argued that an RCT with episiotomy as the intervention in the setting of operative vaginal delivery is impractical. A survey of obstetricians highlighted concerns about the validity of an RCT that evaluates a surgical approach that is not dichotomised into two types of practice, but instead is based on clinical judgement [62]. Sultan et al. [63] provided evidence from observational studies to recommend the liberal use of a MLE/LE cut at 60° during operative vaginal delivery and highlighted further potential limitations of a RCT.

In the absence of an adequately powered RCT, our meta-analysis provides the best available evidence. Our findings are consistent with the RCOG guidance, which recommends that the evidence to support MLE with operative vaginal delivery is stronger for nulliparous women and forceps deliveries [1]. Their evidence for forceps deliveries was based on findings from two large retrospective cohort studies [33, 39]. By completing a meta-analysis, we have statistically pooled together the data from all studies in the literature to generate an overall estimate of the effect of episiotomy with both ventouse and forceps deliveries. However, the inclusion of non-randomised observational studies in our meta-analysis may confer difficulty with precise interpretation of effect size due to low study quality and high risk of bias. We acknowledge that in studies with low/moderate risk of bias, although the incidence of OASI was lower when an MLE/LE was performed with a ventouse or forceps delivery, this reduction did not reach statistical significance. However, in studies with a high/critical risk of bias, a significant clinical benefit was demonstrated. However, no significant sub-group difference was found between studies of low/moderate or high/critical risk of bias. Our results should be interpreted with caution, as routine episiotomy is associated with a significant increase in blood loss, perineal pain, dyspareunia and pelvic floor dysfunction [64]. It is therefore important these risks are considered, including the values and preferences of the woman. However, non-randomised studies may be a better reflection of clinical practice, as intervention choice is at the discretion of the clinician [65, 66].

Parity and instrument type are known significant independent risk factors for OASI, with forceps in particular increasing the odds of OASI six-fold [67]. Therefore, sub-group analysis of these different populations is necessary to evaluate the individual effect size of episiotomy on OASI in at risk groups. Unexpectedly, we found no significant difference between the sub-groups (nulliparous vs. multiparous, forceps vs. ventouse). However, a smaller number of trials and participants contributed data to each subgroup, meaning that the analysis may not be able to detect subgroup differences. Despite sub-group analysis, heterogeneity remained very significant within each sub-group. Two meta-analyses have previously been completed to investigate the effect of MLE with ventouse deliveries and OASI rate [15, 16]. Sagi-Dain et al. [16] concluded from their sample of 290,000 women that, although the incidence of OASI with ventouse delivery was lower with MLE, it was non-significant (OR 68 [95% CI 0.43–1.07]). Lund et al. [15], based on a sample of 320,000 women, found that MLE/LE significantly reduced the odds of OASI by 47% (OR 0.53 [95% CI 0.47–0.77]). However, based on all available evidence to date, our results have demonstrated that with nulliparous women the rate of OASI is significantly reduced by 49% when an MLE/LE is used in ventouse deliveries, which is similar to the findings by Lund et al. [15]. Sagi-Dain et al. [16] also suggested that MLE with ventouse significantly increased the rate of OASI in parous women by 27% (OR 1.27 [95% CI 1.05–1.53]). However, this was not the case with LE, which was analysed separately. In comparison, our review encompassed a larger number of parous women (60,406 women [52,118 = ventouse, 8218 = forceps] vs. 14,640 women). We found that the rate of OASI was lower in multiparous women who had an MLE/LE compared to no episiotomy during a ventouse (2.2% vs. 4.8%) or forceps (4.7% vs. 8.0%) assisted delivery. However, this reduction was not significant. As we included four additional studies and unpublished data from two studies, this strengthens our findings and may also explain the difference in results. It is important to note that number of multiparous women (*n* = 60,406) included in our meta-analysis was much smaller than that of nulliparous women (*n* = 633,089), which is a true reflection of obstetric practice. Moreover, the frequency of OASI was almost twice as high in nulliparous women (6.3%) compared to multiparous women (3.7%). This provides further evidence to explain why MLE/LE was found to be protective with both forceps and ventouse deliveries in nulliparous women compared to multiparous.

## Conclusion

In conclusion, this meta-analysis has shown that MLE/LE is associated with a reduction in the incidence of OASI following operative vaginal delivery, particularly in nulliparous women undergoing a ventouse or forceps assisted delivery. This information will be useful in aiding clinical decision-making and counselling in the antenatal period and during labour. However, the results of this meta-analysis should be interpreted with caution as there was significant unexplained heterogeneity across included studies and the overall quality of evidence was assessed to be very low. Larger, higher quality studies in this area will provide more data to inform future policy.

## Supplementary information

ESM 1 (DOCX 165 kb)
