# Supplementary material for: Mediolateral/lateral episiotomy with operative vaginal delivery and the risk reduction of obstetric anal sphincter injury (OASI): A systematic review and meta-analysis
Source: Int Urogynecol J. 2022 Apr 15;33(6):1393–405. doi: 10.1007/s00192-022-05145-1 (PMC9206628; doi:10.1007/s00192-022-05145-1)
Supplement: Supplementary file 1 — (DOCX 165 kb) [file 192_2022_5145_MOESM1_ESM.docx]

**Electronic Supplementary material (Tables):**

**Figure S1: Funnel plot**

**
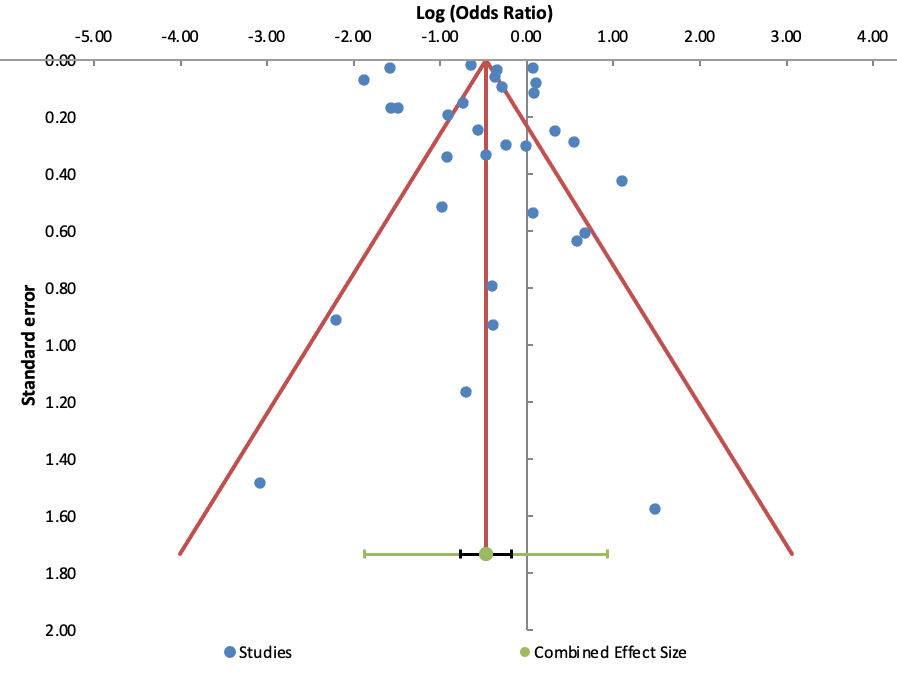
**

**(Table S1)**

Table of excluded studies:

| **Author (Year)** | **Title** | **Journal** | **Reason for Exclusion** | |
| --- | --- | --- | --- | --- |
| Aceituno et al (2011) | Strategy to reduce severe perineal tears during delivery | Clinica e Investigacion en Ginecologia y Obstetricia | Included midline episiotomy | |
| Alouni et al (2011) | Anal sphincter tears after vaginal delivery: risks factors and means of prevention | Revue Médicale de Liège | Results not in line with PICO | |
| Ampt et al (2015) | Obstetric anal sphincter injury rates among primiparous women with different modes of vaginal delivery | International Journal of Gynecology and Obstetrics | Does not state what type of episiotomy used | |
| Anglim et al (2019) | Risk factors and outcome of repair of obstetric anal sphincter injuries as followed up in a dedicated perineal clinic | International Urogynecology Journal | All women included had a history of OASI | |
| Barbier et al (2007) | [Is primiparity, the only risk factor for type 3 and 4 perineal injury, during delivery?]. | La primiparite est-elle le seul facteur de risque des lesions du sphincter anal en cours d'accouchement? | All women undergoing instrumental delivery had an episiotomy | |
| Baskett et al (2008) | A Prospective Observational Study of 1000 Vacuum Assisted Deliveries With the OmniCup Device | Journal of Obstetrics and Gynaecology Canada | Does not state what type of episiotomy used | |
| Baumann et al (2007) | Factors associated with anal sphincter laceration in 40,923 primiparous women. | International Urogynecology Journal | Included midline episiotomy | |
| Bek et al (1991) | Intervention during labor: risk factors associated with complete tear of the anal sphincter | Acta Obstetricia et Gynecologica Scandinavica | Results not in line with PICO | |
| Benifla et al (2000) | Postpartum sphincter rupture and anal incontinence: prospective study with 259 patients | Gynécologie Obstétrique Fertilité . | Unable to obtain full text | |
| Bergendahl et al (2019) | Lateral episiotomy versus no episiotomy to reduce obstetric anal sphincter injury in vacuum-assisted delivery in nulliparous women: study protocol on a randomised controlled trial. | British Medical Journal open | Protocol only |  |
| Borrman et al (2018) | The effects of a severe perineal trauma prevention program in an Australian tertiary hospital: An observational study | Women and Birth | Data not extractable | |
| Bourgon et al (2018) | Obstetrical anal sphincter injuries and vacuum-assisted delivery at term in primiparas | Gynecologie Obstetrique Fertilite et Senologie | Results not in line with PICO | |
| Burrows et al (2004) | Predictors of Third and Fourth-degree Perineal Lacerations | Journal of Pelvic Medicine and Surgery | Does not state what type of episiotomy used | |
| Caudwell et al (2020) | The effect of replacing vacuum with forceps in operative vaginal delivery: an observational study | International Urogynecology Journal | Results not in line with PICO | |
| Chen et al (2019) | Factors associated with obstetric anal sphincter injuries during vacuum delivery among Chinese women | International Journal of Gynecology and Obstetrics | Mediolateral episiotomy excluded | |
| Dahl et al (2006) | Obstetric anal sphincter rupture in older primiparous women: A case-control study | Acta Obstetricia et Gynecologica Scandinavica | Included midline episiotomy | |
| Djakovic et al (2018) | Third and fourth degree perineal tear in four-year period at sestre milosrdnice University hospital center, Zagreb, Croatia | Open Access Macedonian Journal of Medical Sciences | Results not in line with PICO | |
| Fatima et al (2013) | Risk factors for intrapartum perineal tears | Journal of Medical Sciences (Peshawar) | Results not in line with PICO | |
| Feldman et al (1999) | Rotational versus nonrotational forceps: maternal and neonatal outcomes. | American Journal of Obstetrics and Gynecology | Results not in line with PICO | |
| FitzGerald et al (2007) | Risk factors for anal sphincter tear during vaginal delivery | Obstetrics and Gynecology | Included midline episiotomy | |
| Frenette et al (2019) | Impact of Episiotomy During Operative Vaginal Delivery on Obstetrical Anal Sphincter Injuries | Journal of Obstetrics and Gynaecology Canada | Included midline episiotomy | |
| Friedman (2015) | Evaluation of third-degree and fourth-degree laceration rates as quality indicators | Obstetrics and Gynecology | Does not state what type of episiotomy used | |
| Gauthaman et al (2015) | Kielland's forceps: does it increase the risk of anal sphincter injuries? An observational study | International Urogynecology Journal and Pelvic Floor Dysfunction | Does not state what type of episiotomy used | |
| Gebuza et al (2018) | Episiotomy and perineal tear risk factors in a group of 4493 women | Health care for women international | Does not state what type of episiotomy used | |
| Gundabattula et al (2018) | Risk factors for obstetric anal sphincter injuries (OASI) at a tertiary centre in south India | International Urogynecology Journal | Results not in line with PICO | |
| Hauck et al (2015) | Risk factors for severe perineal trauma during vaginal childbirth: A Western Australian retrospective cohort study | Women and Birth | Does not state what type of episiotomy used | |
| Hirsch et al (2018) | Reducing high-order perineal laceration during operative vaginal delivery | American Journal of Obstetrics and Gynecology | Included midline episiotomy | |
| Hudelist et al (2005) | Factors predicting severe perineal trauma during childbirth: Role of forceps delivery routinely combined withMLE | American Journal of Obstetrics and Gynecology | All women undergoing instrumental delivery had an episiotomy | |
| Hudelist et al (2008) | The role of episiotomy in instrumental delivery: is it preventative for severe perineal injury?. | Journal of obstetrics and gynaecology : the journal of the Institute of Obstetrics and Gynaecology | Review article | |
| Jiang et al (2017) | Selective versus routine use of episiotomy for vaginal birth | Cochrane Database of Systematic Reviews | Results not in line with PICO, reference list reviewed and studies were included in our meta-analysis | |
| Kaganova et al (2019) | Analysis of perinatal outcomes and maternal morbidity associated with vacuum-assisted vaginal delivery | Akusherstvo i Ginekologiya (Russian Federation) | Does not state what type of episiotomy used | |
| Karbanova et al (2013) | Mediolateral vs. Lateral episiotomy and associated intrapartal complications in primiparous women | International urogynecology journal and pelvic floor dysfunction | Mediolateral compared to lateral episiotomy | |
| Klein et al (2020) | Routine use of episiotomy with forceps should not be encouraged | Canadian Medical Association Journa | Letter to editor | |
| Kuljak et al (2018) | Third-and fourth-degree perineal tears and restrictive use of episiotomy | Acta Medica Croatica | All women included had a history of OASI | |
| Kwok et al (2019) | Prevalence of obstetric anal sphincter injury following vaginal delivery in primiparous women: A retrospective analysis | Hong Kong Medical Journal | Results not in line with PICO | |
| Levin et al (2019) | Operative delivery in nuliiparous: deserves an episiotomy | Archives of Gynecology and Obstetrics | Letter to editor | |
| Muraca et al (2019) | Episiotomy use among vaginal deliveries and the association with anal sphincter injury: A population-based retrospective cohort study | Canadian Medical Association Journa | Included midline episiotomy | |
| Nettle et al (2019) | Perineal trauma with vaginal birth after a previous caesarean section: A retrospective cohort study | Australian and New Zealand Journal of Obstetrics and Gynaecology | Results not in line with PICO | |
| Pergialiotis et al (2014) | Risk factors for severe perineal lacerations during childbirth | International Journal of Gynecology and Obstetrics | Results not in line with PICO, reference list reviewed and studies were included in our meta-analysis | |
| Pergialiotis et al (2020) | Risk factors for severe perineal trauma during childbirth: An updated meta-analysis | European Journal of Obstetrics and Gynecology and Reproductive Biology | Results not in line with PICO, reference list reviewed and studies were included in our meta-analysis | |
| Poen et al (1997) | Third degree obstetric perineal tears: risk factors and the preventive role ofMLE. | BJOG: An International Journal of Obstetrics and Gynaecology, | Data not extractable | |
| Ramm et al (2018) | Risk factors for the development of obstetric anal sphincter injuries in modern obstetric practice | Obstetrics and Gynecology | Included midline episiotomy | |
| Reinbold et al (2012) | [From the impact of French guidelines to reduce episiotomy's rate]. | De l'impact des RPC pour reduire le taux d'episiotomie. | Does not state what type of episiotomy used | |
| Revicky et al (2010) | Could aMLE prevent obstetric anal sphincter injury? | European Journal of Obstetrics and Gynecology and Reproductive Biology | Results not in line with PICO | |
| Rezaie et al (2014) | Comparison of perineal lacerations in routine vs. SelectiveMLE among women referring to the obstetrics department of besat hospital in sanandaj in 2011 | Iranian journal of obstetrics, gynecology and infertility | Unable to get translation of paper | |
| Robinson et al (1999) | Episiotomy, operative vaginal delivery, and significant perinatal trauma in nulliparous women | American Journal of Obstetrics and Gynecology | Included midline episiotomy | |
| Rodriguez-Colorado et al (2019) | Risk factors associated with obstetric anal sphincter injury: A retrospective cohort | Ginecologia y Obstetricia de Mexico | Results not in line with PICO | |
| Sagi-Dain et al (2017) | No episiotomy versus selective lateral/mediolateral episiotomy (EPITRIAL): an interim analysis | International Urogynecology Journal | Full analysis included in meta-analysis | |
| Segal et al (2020) | Risk factors for obstetric anal sphincter injuries (OASIS) during vacuum extraction delivery in a university affiliated maternity hospital | Journal of Maternal-Fetal and Neonatal Medicine | Does not state what type of episiotomy used | |
| Simic et al (2017) | Duration of second stage of labor and instrumental delivery as risk factors for severe perineal lacerations: population-based study. | BMC pregnancy and childbirth | Included midline episiotomy | |
| Sooklim et al (2007) | The outcomes of midline versus medio-lateral episiotomy | Reproductive Health | Results not in line with PICO | |
| Sultan et al (1994) | Third degree obstetric anal sphincter tears: risk factors and outcome of primary repair | British Medical Journal | Results not in line with PICO | |
| Wood et al (1998) | Third degree anal sphincter tears: risk factors and outcome | Australia and New Zealand Journal of Obstetrics and Gynaecology | Does not state what type of episiotomy used, all women undergoing forceps delivery had an episiotomy | |
| Zhu et al (2015) | Restrictive use of episiotomy for low forceps delivery | National Medical Journal of China | Data not extractable | |
| Grier et al (1948) | Prophylactic low forceps and episiotomy | Journal of Omaha Midwest Clinical Society | Unable to obtain full text | |
| Hafeez et al (2013) | Indications and risks of vacuum assisted deliveries | Journal International Medical Sciences Academy | Unable to obtain full text | |

**(Table S2)**

Risk of bias of the randomised studies included in the meta-analysis

|  | **R** | **ASI** | **AI** | **DI** | **MO** | **RR** |
| --- | --- | --- | --- | --- | --- | --- |
| **Murphy et al** [25] | **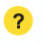** | **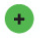** | **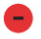** | **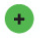** | **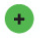** | **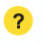** |
| **Sagi Dain et al** [26] | **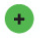** | **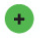** | **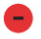** | **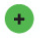** | **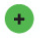** | **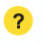** |

R- Randomization

ASI- Assignment to intervention

AI- Adhering to intervention

DI-Deviations from the intended interventions

MO- Measurement of the outcome

RR- Reported results

**
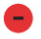
**= high risk of bias

**
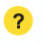
** = moderate risk of bias

**
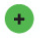
** =low risk of bias

**(Table S3):**

Risk of bias of the non-randomised studies included in the meta-analysis

| **Authors** | **C** | **S** | **CI** | **DI** | **MD** | **O** | **R** |
| --- | --- | --- | --- | --- | --- | --- | --- |
| **Ampt et al** [54] | **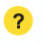** | **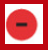** | **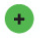** | **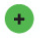** | **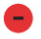** | **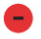** | **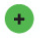** |
| **Aukee et al** [34] | **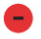** | **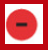** | **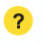** | **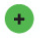** | **-** | **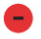** | **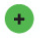** |
| **Baghurst et al** [35] | **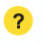** | **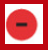** | **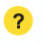** | **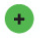** | **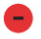** | **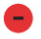** | **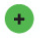** |
| **Bodner-Adler et al** [36] | **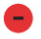** | **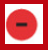** | **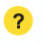** | **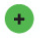** | **-** | **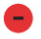** | **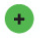** |
| **Boujenah et al** [37] | **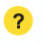** | **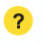** | **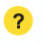** | **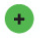** | **-** | **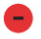** | **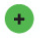** |
| **D'Souza et al** [38] | **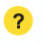** | **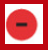** | **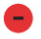** | **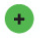** | **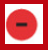** | **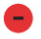** | **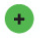** |
| **De Leeuw et al** [39] | **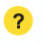** | **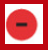** | **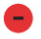** | **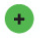** | **-** | **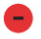** | **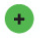** |
| **De Parades et al** [28] | **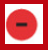** | **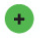** | **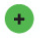** | **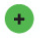** | **-** | **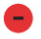** | **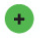** |
| **De Vogel et al** [40] | **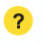** | **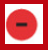** | **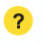** | **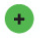** | **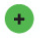** | **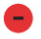** | **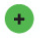** |
| **Gachon et al** [41] | **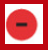** | **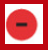** | **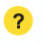** | **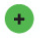** | **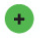** | **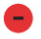** | **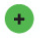** |
| **Hamouda et al** [27] | **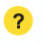** | **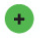** | **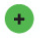** | **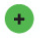** | **-** | **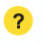** | **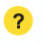** |
| **Gurol-Urganci et al** [33] | **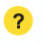** | **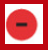** | **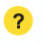** | **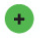** | **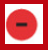** | **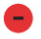** | **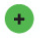** |
| **Jango et al** [42] | **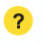** | **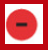** | **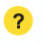** | **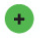** | **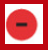** | **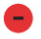** |  |
| **Levin et al** [43] |  |  |  |  | **-** |  |  |
| **Macleod et al** [29] |  |  |  |  | **-** |  |  |
| **Marschalek et al** [44] |  |  |  |  | **-** |  |  |
| **Meyer et al** [53] |  |  |  |  | **-** |  |  |
| **Parnell et al** [30] |  |  |  |  | **-** |  |  |
| **Räisänen et al** [45] |  |  |  |  |  |  |  |
| **Räisänen et al** [46] |  |  |  |  | **-** |  |  |
| **Rognant et al** [47] |  |  |  |  |  |  |  |
| **Rygh et al** [31] |  |  |  |  |  |  |  |
| **Schmitz et al** [48] |  |  |  |  |  |  |  |
| **Shmueli et al** [49] |  |  |  |  | **-** |  |  |
| **Van Bavel et al**[50] |  |  |  |  |  |  |  |
| **Yamasato et al** [51] |  |  |  |  | **-** |  |  |
| **Youssef et al**[42] |  |  |  |  | **-** |  |  |
| **Van Roon et al** [32] |  |  |  |  | **-** |  |  |
| **Vathanan et al** [55] |  |  |  |  |  |  |  |

C= Confounding

S= Selection of participants

CI= Classification of interventions

DI= Deviations from intended interventions

MD= Missing data

O= Outcome measurement

R= Result reporting

= Critical risk of bias

= Serious risk of bias

= Moderate risk of bias

= Low risk of bias

= No information

**Supplementary material:** **Appendix S1**

MOOSE checklist

| **Item number** | **Recommendation** | **Reported on page** |
| --- | --- | --- |
| **Background** | | |
| 1 | Problem definition | To investigate the effectiveness of mediolateral or lateral episiotomy use with instrumental delivery in the prevention of OASI (page 5) |
| 2 | Hypothesis statement | The Royal College of Obstetricians and Gynaecologists (RCOG) Green Top Guideline suggests that in the absence of robust evidence to support either routine or restrictive use of episiotomy at assisted vaginal birth, the decision should be tailored to the circumstances at the time and the preferences of the woman. The evidence to support use ofMLE at assisted vaginal birth in terms of preventing OASI is stronger for nulliparous women and for birth via forceps (page 5) |
| 3 | Description of study outcome(s) | The primary outcome of this study was the Risk of developing obstetric anal sphincter injury (OASI) (page 6) |
| 4 | Type of exposure or intervention used | Eligible studies had to analyse the risk of OASI in assisted vaginal birth with and without mediolateral or lateral episiotomy (page 6) |
| 5 | Type of study design used | Randomised controlled trials (RCTs), non-randomised controlled trials, prospective and retrospective observational studies (page 6) |
| 6 | Study population | Women undergoing operative vaginal delivery (page 6) |
| **Search Strategy** | | |
| 7 | Qualifications of searchers | Credentials are listed on the title page |
| 8 | Search strategy, including time period included in the synthesis and key words | Search strategy and selection criteria (page 6) and Appendix S1 |
| 9 | Effort to include all available studies, including contact with authors | Authors of included studies were contacted if the full text could not be retrieved.  Other relevant systematic reviews of MLE/LE episiotomy with operative vaginal delivery and the reference lists of the eligible studies were also searched (page 7) |
| 10 | Databases and registries searched | OVID Medline, Embase and the Cochrane library (page 6) |
| 11 | Search software used, name and version, including special features used | Zotero reference management system (page 6) |
| 12 | Use of hand searching | Other relevant systematic reviews of MLE/LE episiotomy with operative vaginal delivery and the reference lists of the eligible studies were also searched (page 7) |
| 13 | Lists of citations located and those excluded, including justification | Details of the literature search are described in the PRISMA flow chart (Figure 1) |
| 14 | Method of addressing articles published in languages other than English | Acknowledgments (page 14) |
| 15 | Method of handling abstracts and unpublished studies | Case reports, case series, narrative reviews and conference abstracts were excluded (page 6) |
| 16 | Description of any contact with authors | Authors of included studies were contacted if the full text could not be retrieved. In addition, if data reported was incomplete, unclear or published in a manner that was not extractable (page 7). Authors who responded and provided unpublished data were acknowledged (page 14) |
| **Reporting of methods** | | |
| 17 | Description of relevance or appropriateness of studies assembled for assessing the hypothesis to be tested | Search strategy and selection criteria (page 6) and Appendix S1 |
| 18 | Rationale for the selection and coding of data | Data extraction paragraph (page 6-7). Data relevant to the population was extracted including study characteristics, parity, type of operative vaginal delivery, type of episiotomy and rate of OASI. |
| 19 | Documentation of how data were classified and coded | Data extraction paragraph (page 6-7). |
| 20 | Assessment of confounding | We extracted information regarding confounders consistently used in each study. Therefore, separate analysis for parity and instrument type was completed (Table 1) |
| 21 | Assessment of study quality, including blinding of quality assessors, stratification or regression on possible predictors of study results | Two reviewers independently assessed the overall quality of the evidence using criteria recommended by the Grading of Recommendations Assessment, Development and Evaluation working group (GRADE) (page 7,8) |
| 22 | Assessment of heterogeneity | The heterogeneity among studies was calculated using the I^2^ statistic (page 7) |
| 23 | Description of statistical methods in sufficient detail to be replicated | The random-effects model was used if heterogeneity was significant (I^2^ > 50 %) (page 7) |
| 24 | Provision of appropriate table and graphics | We provided the PRISMA flow-chart, included/excluded study characteristics tables and forest plots |
| **Results** | | |
| 25 | Graphic summarising individual study estimates and overall estimate | **Table 1, Figure 1,2,3, S** |
| 26 | Table giving descriptive information for each study included | **Table 1** |
| 27 | Results of sensitivity testing | **Figure 1, 2,** |
| 28 | Indication of statistical uncertainty of findings | 95% confidence intervals were presented with all odd ratios and I2 values |
| **Discussion** | | |
| 29 | Quantitative assessment of bias | Risk of bias assessment of RCTs was conducted using the Cochrane risk-of-bias tool for randomized trials (RoB 2). Non-randomised studies including observational studies were assessed using the Risk Of Bias in Non-randomized Studies - of Interventions (ROBINS-I) tool. (page 7) |
| 30 | Justification for exclusion | Summary of excluded study table (Table |
| 31 | Assessment of quality of included studies | Two reviewers independently assessed the overall quality of the evidence using criteria recommended by the Grading of Recommendations Assessment, Development and Evaluation working group (GRADE) (page 7,8) |
| **Conclusions** | | |
| 32 | Consideration of alternative explanation for observed results | Limitations are discussed on page 12-13. Most important include, the inclusion of non-randomised studies and being unable to control for the angle of episiotomy in 29/30 studies |
| 33 | Generalisation of the conclusions | Mediolateral or lateral episiotomy reduces the risk of OASI in operative vaginal delivery, particularly in nulliparous women undergoing a ventouse or forceps assisted delivery (page 14) |
| 34 | Guidelines for future research | In the absence of an adequately powered RCT, our meta-analysis is the best available evidence, until further studies are completed (page 13) |
| 35 | Disclosure of funding source | No funding required |

**Supplementary material: Appendix S1**

Database: Embase <1974 to 2020 June 24>

Search Strategy:

--------------------------------------------------------------------------------

1 (Obstetric anal sphincter injur* or OASIS or obstetric anal sphincter trauma or

obstetrical injury to the anal sphincter or third-degree perineal laceration* or third

degree perineal laceration* or third-degree tear* or third degree tear* or fourthdegree

laceration* or fourth degree laceration* or fourth-degree tear* or fourth degree

tear* or severe perineal trauma or third-degree perineal tear* or third degree perineal

tear* of fourth-degree perineal tear* or fourth degree perineal tear*).mp. [mp=title,

abstract, heading word, drug trade name, original title, device manufacturer, drug

manufacturer, device trade name, keyword, floating subheading word, candidate

term word] (5865)

2 exp anus injury/ or exp anus sphincter/ or exp anus sphincter disorder/ (10466)

3 exp episiotomy/ (4510)

4 1 or 2 or 3 (18833)

5 (Mediolateral Episiotom* or medio-lateral episiotom* or midline episiotom* or

median epistom* or mediolateral incision or Perineotomy).mp. [mp=title, abstract,

heading word, drug trade name, original title, device manufacturer, drug

manufacturer, device trade name, keyword, floating subheading word, candidate

term word] (471)

6 perineum injury/ or episiotomy/ or anus sphincter/ (14825)

7 5 or 6 (14868)

8 (instrumental deliver* or assisted vaginal deliver* or assisted vaginal birth* or

operative vaginal deliver* or assisted birth* or assisted deliver*).mp. [mp=title,

abstract, heading word, drug trade name, original title, device manufacturer, drug

manufacturer, device trade name, keyword, floating subheading word, candidate

term word] (7588)

9 (forcep* or forceps delivery or clamp).mp. [mp=title, abstract, heading word, drug

trade name, original title, device manufacturer, drug manufacturer, device trade

name, keyword, floating subheading word, candidate term word] (129713)

10 exp obstetric forceps/ or exp fetal extraction device/ (1157)

11 forceps delivery/ (3138)

12 9 or 10 or 11 (130540)

13 (vacuum delivery or vacuum extract* or vacuum-assisted vaginal delivery or

vacuum assisted vaginal delivery or vacuum assisted delivery or vacuum-assisted

delivery or ventouse).mp. [mp=title, abstract, heading word, drug trade name, original

title, device manufacturer, drug manufacturer, device trade name, keyword, floating

subheading word, candidate term word] (4029)

14 exp vacuum extraction/ (2745)

15 13 or 14 (4029)

16 4 and 7 and 12 (968)

17 4 and 7 and 15 (573)

18 4 and 7 and 12 and 15 (391)

19 4 and 7 and 8 and 12 and 15 (238)

Database: Ovid MEDLINE(R) and Epub Ahead of Print, In-Process & Other Non-

Indexed Citations and Daily <1946 to June 24, 2020>

Search Strategy:

--------------------------------------------------------------------------------

1 (Obstetric anal sphincter injur* or OASIS or obstetric anal sphincter trauma or

obstetrical injury to the anal sphincter or third-degree perineal laceration* or third

degree perineal laceration* or third-degree tear* or third degree tear* or fourthdegree

laceration* or fourth degree laceration* or fourth-degree tear* or fourth degree

tear* or severe perineal trauma or third-degree perineal tear* or third degree perineal

tear* of fourth-degree perineal tear* or fourth degree perineal tear*).mp. [mp=title,

abstract, original title, name of substance word, subject heading word, floating subheading

word, keyword heading word, organism supplementary concept word,

protocol supplementary concept word, rare disease supplementary concept word,

unique identifier, synonyms] (3963)

2 delivery, obstetric/ or exp episiotomy/ (30880)

3 1 or 2 (34286)

4 (Mediolateral Episiotom* or medio-lateral episiotom* or midline episiotom* or

median epistom* or mediolateral incision or Perineotomy).mp. [mp=title, abstract,

original title, name of substance word, subject heading word, floating sub-heading

word, keyword heading word, organism supplementary concept word, protocol

supplementary concept word, rare disease supplementary concept word, unique

identifier, synonyms] (330)

5 exp Episiotomy/ (2168)

6 4 or 5 (2266)

7 (instrumental deliver* or assisted vaginal deliver* or assisted vaginal birth* or

operative vaginal deliver* or assisted birth* or assisted deliver*).mp. [mp=title,

abstract, original title, name of substance word, subject heading word, floating subheading

word, keyword heading word, organism supplementary concept word,

protocol supplementary concept word, rare disease supplementary concept word,

unique identifier, synonyms] (3305)

8 (forcep* or forceps delivery or clamp).mp. [mp=title, abstract, original title, name

of substance word, subject heading word, floating sub-heading word, keyword

heading word, organism supplementary concept word, protocol supplementary

concept word, rare disease supplementary concept word, unique identifier,

synonyms] (99766)

9 exp surgical instruments/ or exp obstetrical forceps/ (24384)

10 8 or 9 (120158)

11 (vacuum delivery or vacuum extract* or vacuum-assisted vaginal delivery or

vacuum assisted vaginal delivery or vacuum assisted delivery or vacuum-assisted

delivery or ventouse).mp. [mp=title, abstract, original title, name of substance word,

subject heading word, floating sub-heading word, keyword heading word, organism

supplementary concept word, protocol supplementary concept word, rare disease

supplementary concept word, unique identifier, synonyms] (2775)

12 exp Vacuum Extraction, Obstetrical/ (1313)

13 11 or 12 (2775)

14 3 and 6 and 10 (260)

15 3 and 6 and 13 (155)

16 3 and 6 and 10 and 13 (107)

17 3 and 6 and 7 and 10 and 13 (60)

Search Name: cochrane final search

Date Run: 24/06/2020

--------------------------------------------------------------------------------

ID Search Hits

#1 Obstetric anal sphincter injur* or OASIS or obstetric anal sphincter trauma or obstetrical injury to the anal sphincter or third-degree perineal laceration* or third degree perineal laceration* or third-degree tear* or third degree tear* or fourth-degree laceration* or fourth degree laceration* or fourth-degree tear* or fourth degree tear* or severe perineal trauma or third-degree perineal tear* or third degree perineal tear* of fourth-degree perineal tear* or fourth degree perineal tear* 1328

#2 Mediolateral Episiotom* or medio-lateral episiotom* or midline episiotom* or median epistom* or mediolateral incision or Perineotomy 266

#3 MeSH descriptor: [Episiotomy] explode all trees 261

#4 #2 or #3 487

#5 instrumental deliver* or assisted vaginal deliver* or assisted vaginal birth* or operative vaginal deliver* or assisted birth* or assisted deliver* 6308

#6 forcep* or forceps delivery or clamp 6621

#7 MeSH descriptor: [Surgical Instruments] explode all trees 748

#8 MeSH descriptor: [Obstetrical Forceps] explode all trees 50

#9 #6 or #7 or #8 7217

#10 vacuum delivery or vacuum extract* or vacuum-assisted vaginal delivery or vacuum assisted vaginal delivery or vacuum assisted delivery or vacuum-assisted delivery or ventouse 1145

#11 MeSH descriptor: [Vacuum Extraction, Obstetrical] explode all trees 81

#12 #10 or #11 1145

#13 #1 and #4 and #9 30

#14 #1 and #4 and #12 21

#15 #1 and #4 and #9 and #12 18

#16 #1 and #4 and #5 and #9 and #12 18

#17 #1 and #4 and #5 and #9 26

#18 #1 and #4 and #5 and #12 20
